# Polynucleotide (PN) Therapy for Accelerated Wound Repair and Scar Modulation in Aesthetic Procedures

**DOI:** 10.1007/s00266-026-05843-x

**Published:** 2026-05-20

**Authors:** Soo-Yeon Park, Michael Kim, Song-Eun Yoon, Jong-Keun Song, Jake Sloane, Jeremy B. Green, Thomas Rappl, Jin-Hyun Kim, Kyu-Ho Yi

**Affiliations:** 1 Made-Young Plastic Surgery Clinic, Seoul, Republic of Korea; 2 Pharmaresearch Co., Ltd. Integrated R&D Center, 22, Geumto-ro 40beon-gil, Seongnam, Republic of Korea; 3 Brandnew Aesthetic Surgery Clinic, Seoul, Republic of Korea; 4 Pixelab Plastic Surgery Clinic, Seoul, Republic of Korea; 5 Infinity Skin Clinic, Surry Hills, NSW Australia; 6 Skin Associates of South Florida/Skin Research Institute Coral Gables, Coral Gables, FL USA; 7 Medical Aesthetic Research Academy, Graz, Austria; 8 You and I Clinic, Seoul, Republic of Korea

**Keywords:** Polynucleotide, Wound healing, Scar modulation, Facelift, Aesthetic surgery

## Abstract

**Background:**

Postoperative wound compromise after aesthetic procedures may manifest as delayed epithelialization, tissue ischemia, hypertrophic scarring, contour depression, or wound dehiscence. These problems are more common in patients with impaired microcirculation or other healing-related comorbidities.

**Objectives:**

To describe the clinical use of injectable polynucleotide as an adjunctive treatment for postoperative wound healing and scar modulation after aesthetic procedures.

**Methods:**

This retrospective case series included eight consecutive patients treated in routine practice at a single aesthetic clinic over a continuous study accrual period. Eligible patients had clinically significant wound healing delay, early ischemic change, hypertrophic scarring, or postoperative contour depression after aesthetic surgery or facial trauma and were managed with injectable polynucleotide (Rejuran). Treatments were individualized according to wound status and tissue quality, typically using 1–2 mL per session at 2- to 3-week intervals. Because of the heterogeneous case mix and retrospective design, outcomes were evaluated by serial standardized clinical photographs and physician-rated changes in wound size, erythema, scar width/elevation, and contour rather than by a single validated scar scale.

**Results:**

All eight patients demonstrated clinical improvement after treatment, including faster re-epithelialization, reduction of erythema, narrowing and flattening of scars, improved contour, or complete wound closure. Earlier treatment during the inflammatory or early proliferative phase appeared to produce the most favorable results. No treatment-related adverse events were documented.

**Conclusions:**

The application of injectable polynucleotide therapy appears to be a safe and useful adjunct for postoperative wound care and scar modulation in aesthetic practice. Early use may be especially helpful in patients at high risk for wound breakdown, although prospective studies using standardized outcome measures are needed.

**Level of Evidence V:**

This journal requires that authors assign a level of evidence to each article. For a full description of these Evidence-Based Medicine ratings, please refer to the Table of Contents or the online Instructions to Authors www.springer.com/00266.

## Introduction

Optimal wound healing and scar quality are central to successful aesthetic surgery. Even technically well-performed procedures can be compromised by flap ischemia, wound maceration, dehiscence, hypertrophic scarring, or localized soft-tissue depression. Such problems are particularly challenging in patients with diabetes mellitus, hypertension, smoking history, or other factors that may impair perfusion and tissue remodeling.

Biological interest in nucleic acid–based biomolecules is rooted in the broader role of nucleic acids as informational and regulatory macromolecules [1]. Polynucleotide (PN) and polydeoxyribonucleotide (PDRN) products are DNA-derived biomaterials used in regenerative medicine and aesthetic practice. Although the terminology has sometimes been used inconsistently in the literature, PN and PDRN should be distinguished in terms of regulatory classification and primary mode of action. PDRN is typically discussed as a pharmaceutical agent with pharmacologic activity, whereas PN products used in aesthetic practice, such as the long-chain injectable formulation used in our cases (Rejuran), are better characterized as medical devices that provide three-dimensional scaffold support for tissue restoration [2, 3]. In this manuscript, PN is used as the principal term for the injectable product administered in our cases.

Clinical evidence relevant to wound healing and scar modulation is accumulating. Prior reports have described benefits of PDRN in venous ulcer healing and tissue regeneration [4, 5], while PN has been widely utilized for skin rejuvenation [6], post-surgical scar prevention [7], and managing aesthetic soft-tissue quality through physical support [8]. These data provide a rationale for evaluating injectable PN as a postoperative adjunct in aesthetic surgery, especially in patients who show early evidence of compromised healing or abnormal scar evolution.

The purpose of this retrospective case series was therefore to describe the real-world use of injectable PN therapy across a spectrum of postoperative wound healing and scar-related problems encountered after aesthetic procedures.

## Methods

This was a retrospective case series of eight consecutive patients treated at a single aesthetic practice over a continuous routine practice accrual period. Consecutive inclusion was used to reduce selection bias: All patients who met the clinical criteria for PN treatment during the study period and for whom adequate clinical photographs and follow-up documentation were available were reviewed for inclusion. The series included patients with postoperative wound delay, early ischemic change, wound separation, hypertrophic scarring, or contour depression after facelift-related procedures, as well as one patient with a traumatic upper-lip scar.

Inclusion criteria were: (1) A clinically significant wound healing or scar-related problem judged by the treating physician to warrant adjunctive PN therapy; (2) available serial clinical photographs before and after treatment; and (3) follow-up sufficient to document short-term clinical response. Patients with uncontrolled active infection, known hypersensitivity to PN products, or inadequate follow-up documentation were excluded.

The injectable product used in all cases was a polynucleotide preparation. PN was administered intradermally or into the superficial subdermal plane using a fine needle, with attention to intradermal injection depth and the current classification of PN products as skin boosters [8, 9]. Most patients received one to three sessions separated by approximately 2–3 weeks. Earlier treatment was favored when wound maceration, ischemic discoloration, or early scar thickening was observed.

Because this series included heterogeneous clinical presentations, no single validated scar scale had been prospectively applied at the time of care. Outcomes were therefore assessed retrospectively using serial standardized clinical photographs and physician evaluation of changes in wound size, epithelialization, erythema, scar width/elevation, and contour restoration. This limitation has been made explicit because the results rely primarily on clinical observation and photographic comparison.

Safety was assessed at each follow-up visit by documenting adverse events, including worsening inflammation, nodularity, infection, allergic reaction, or other procedure-related complications.

Ethics statement: This retrospective review involved treatments delivered as part of routine clinical care. The manuscript was prepared from de-identified clinical records and photographs obtained during standard management, and all patients provided written informed consent for treatment and clinical photography. Because this report describes a retrospective, non-interventional case series based on anonymized routine practice data, no separate institutional review board approval number was available for the present analysis.

## Cases

### Case 1. Forehead Burn Injury After Endoscopic Forehead Lift

A patient developed a forehead burn after an endoscopic forehead lift, likely related to pressure ischemia from postoperative dressing or compromise of the dermal plexus. Instead of surgical revision, PN was injected around the wound margin. Serial photographs showed progressive re-epithelialization, reduction of inflammation, and eventual healing with limited residual discoloration (Fig. 1).

**Fig. 1:**
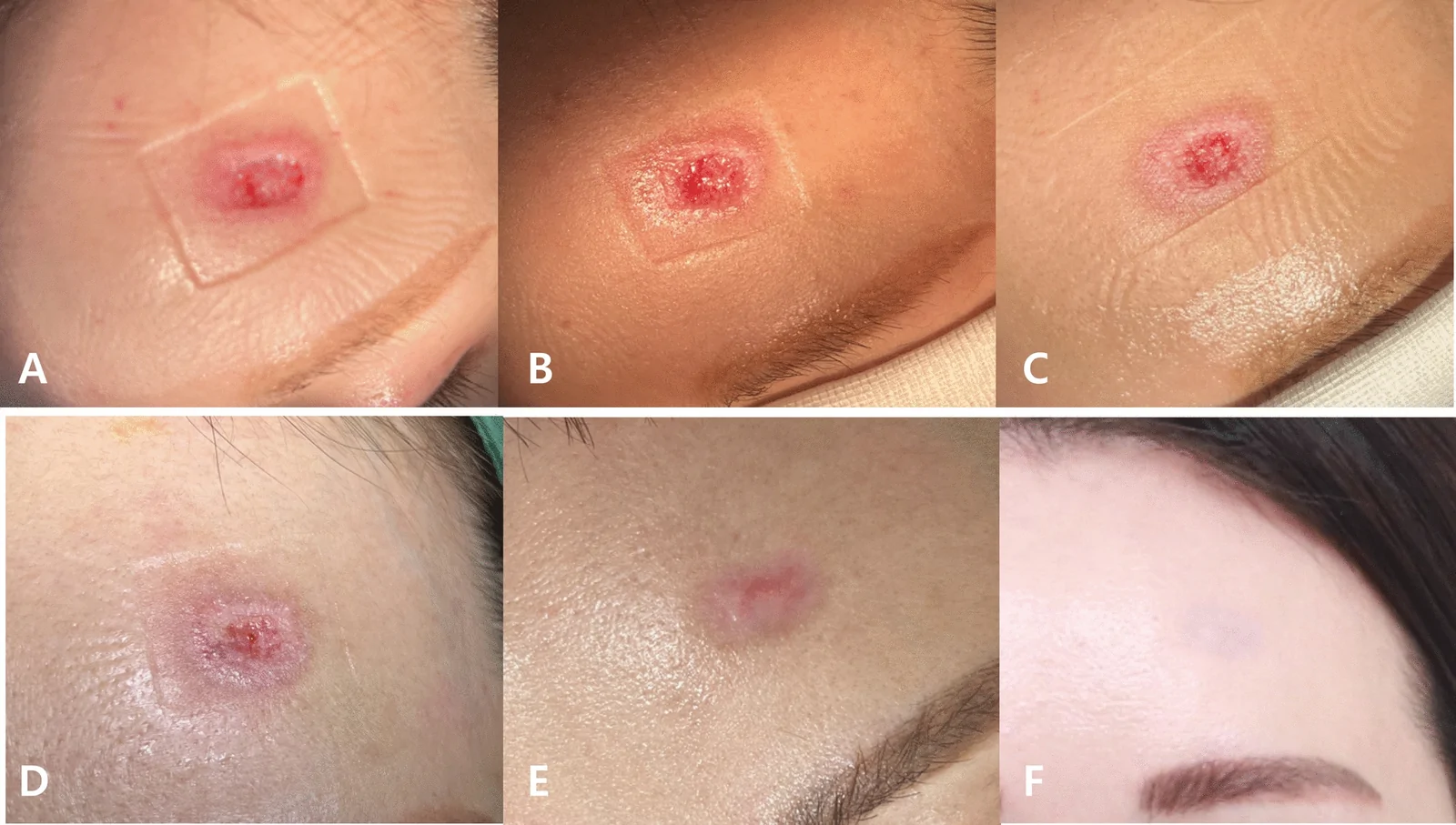
Sequential healing of a forehead burn/ulcer after PN treatment, demonstrating progressive epithelialization and limited residual discoloration

### Case 2. Post-Facelift Soft-Tissue Depression

Six months after facelift surgery, a patient presented with a visible contour depression in the periauricular area. Three PN sessions (1 mL each at 3-week intervals) were performed intradermally. Follow-up photographs demonstrated partial restoration of soft-tissue volume and improved contour symmetry (Fig. 2).

**Fig. 2:**
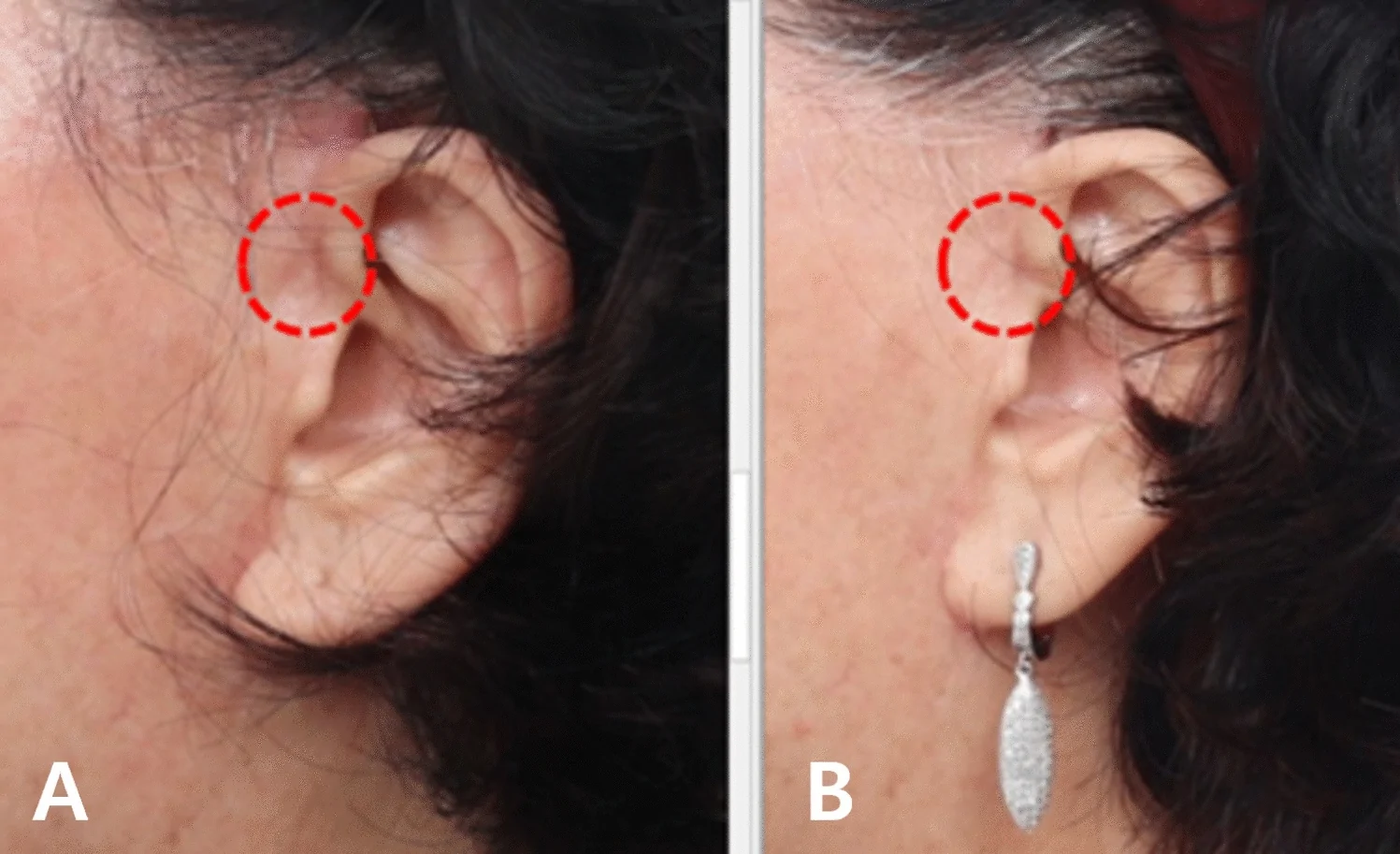
Improvement of postoperative periauricular soft-tissue depression after serial DOT-PN injections

### Case 3. Mature Hypertrophic Post-Facelift Scar

A patient presented six months after facelift surgery with a thickened, elevated, erythematous scar behind the ear. PN therapy was administered in three sessions (2 mL each at 3-week intervals). The scar became flatter, less erythematous, and texturally smoother on follow-up examination (Fig. 3).

**Fig. 3:**
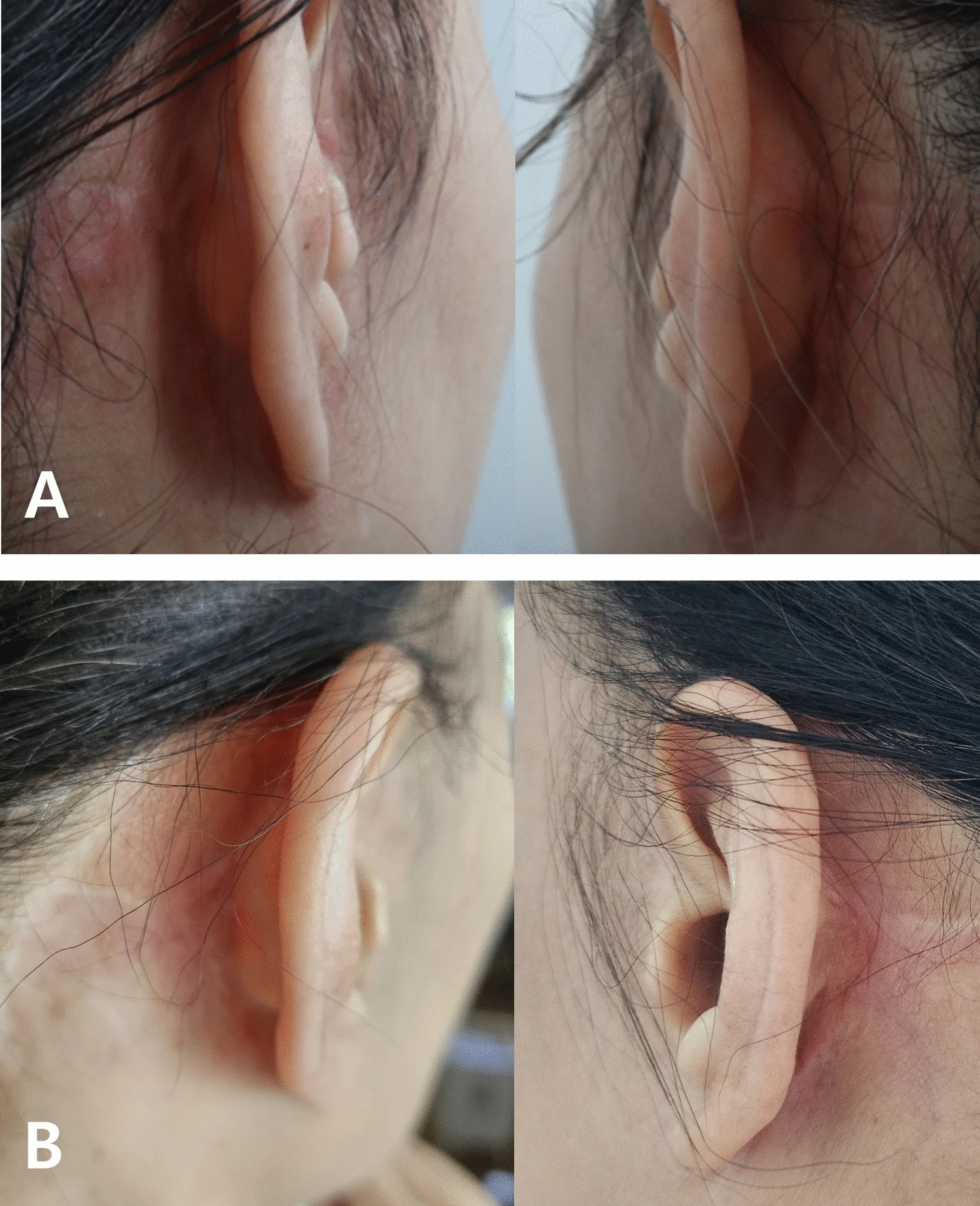
Flattening and decreased erythema of a mature hypertrophic scar behind the ear after PN therapy

### Case 4. Delayed Wound Maturation in a Patient with Diabetes Mellitus

A patient with diabetes mellitus showed prolonged wound maceration and delayed maturation around the periauricular facelift incision. A single 2-mL PN treatment was administered. Three-week follow-up showed progressive remodeling, decreased pigmentation change, and improved local tissue quality (Fig. 4).

**Fig. 4:**
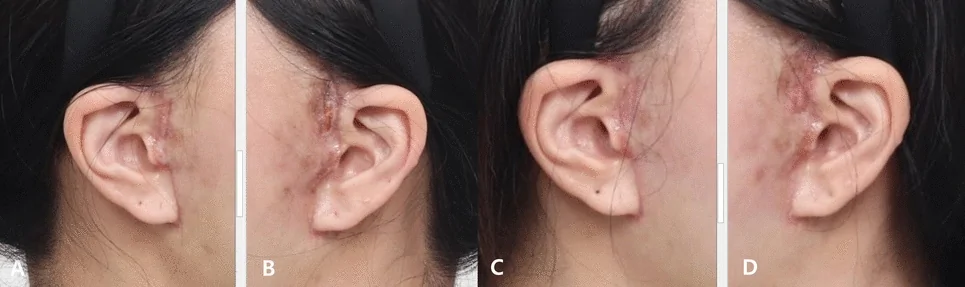
Remodeling of a hypertrophic preauricular facelift scar after serial PN treatment

### Case 5. Upper-Lip Traumatic Scar

A patient with a traumatic upper-lip scar showed mild hypertrophy, redness, and textural irregularity six months after injury. One intradermal PN treatment (1 mL) was given. Follow-up photographs demonstrated reduced erythema and flattening of the scar (Fig. 5).

**Fig. 5:**
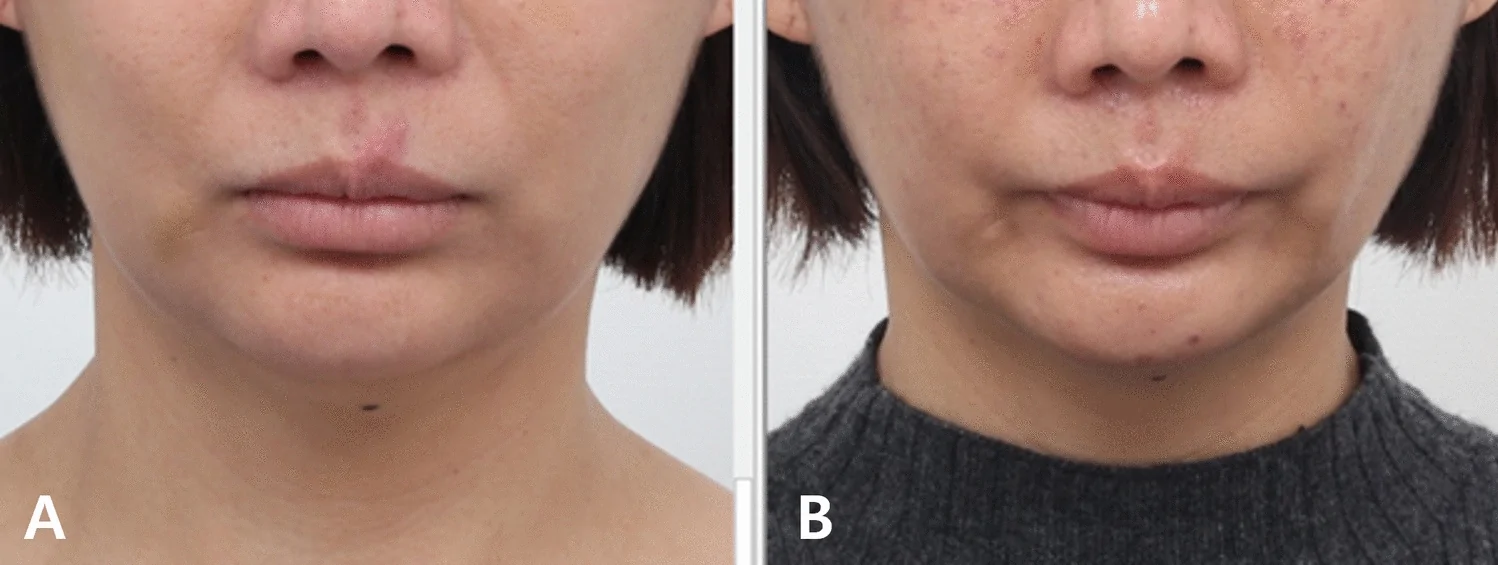
Improvement of a traumatic upper-lip scar after a single PN treatment

### Case 6. Early Wound Maceration and Eschar Formation After Facelift in a High-Risk Patient

Seven days after facelift surgery and suture removal, a patient with diabetes and hypertension showed wound maceration and early eschar formation, indicating a risk of necrosis and poor scar evolution. PN (2 mL) was started immediately and repeated twice at 3-week intervals. The wound progressively stabilized, re-epithelialized, and healed without secondary contour deformity (Fig. 6).

**Fig. 6:**
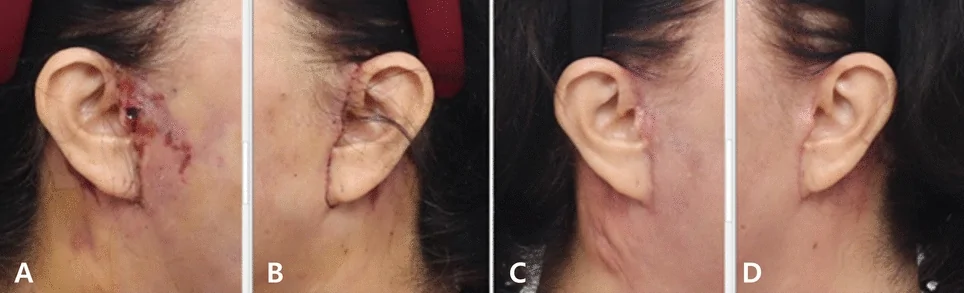
Healing of macerated, high-risk facelift wounds in a patient with diabetes mellitus and hypertension after serial PN sessions

### Case 7. Early Tragal Wound Dehiscence in a Patient with Diabetes and Hypertension

During the first postoperative week, a patient developed a wound defect near the tragus with exposure of vulnerable tissue and concern for contour distortion. PN (2 mL) was administered at stitch removal with two additional sessions over the next 6 weeks. The wound narrowed and healed while preserving tragal contour (Fig. 7).

**Fig. 7:**
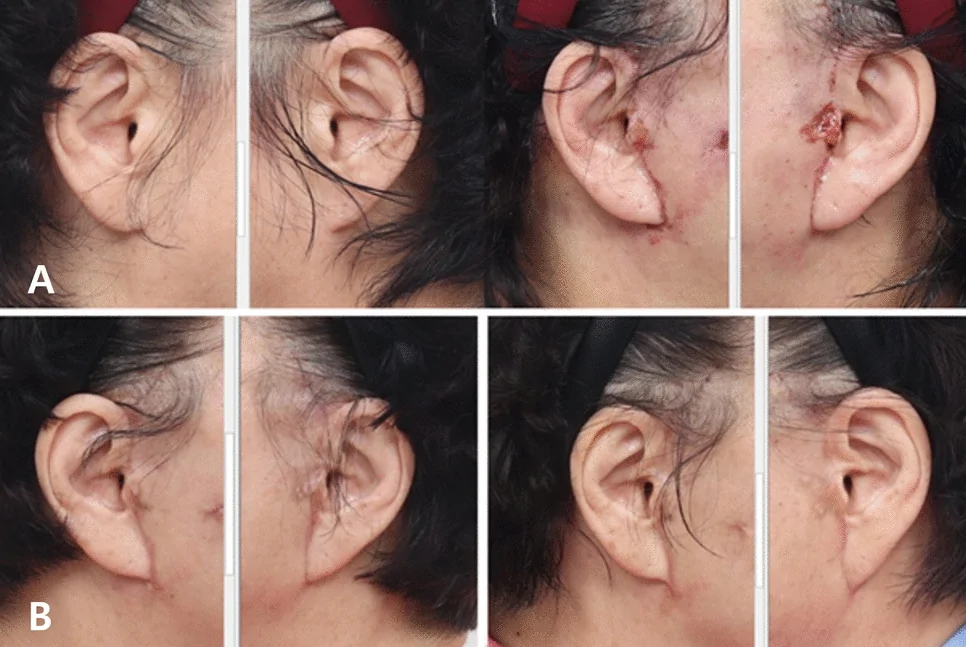
Serial progression of early postoperative wound compromise and subsequent healing after PN treatment

### Case 8. Wound Separation at the Tragal Margin Two Weeks After Surgery

Two weeks after facelift surgery, a patient showed slight wound separation and persistent erythema anterior to the tragus. A single 2-mL DOT-PN treatment was administered. On follow-up, wound closure was complete and periwound redness had resolved (Fig. 8).

**Fig. 8:**
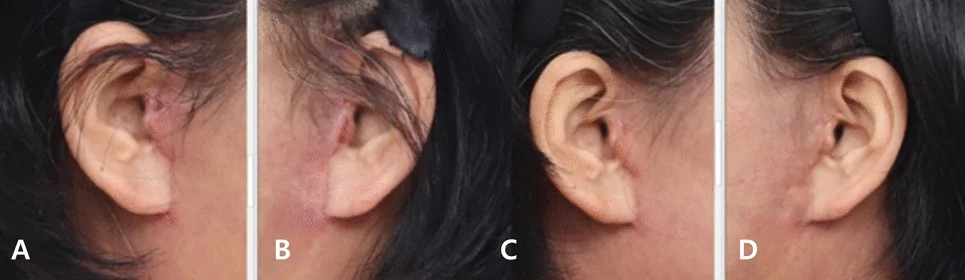
Closure and scar improvement of tragal-margin wound separation after PN treatment

## Discussion

This case series suggests that injectable PN therapy may be useful across several postoperative scenarios relevant to aesthetic surgery: early ischemic change, delayed epithelialization, wound separation, hypertrophic scarring, and localized contour depression. Across all eight cases, improvement was documented by serial photographs and clinical follow-up, with no treatment-related adverse events.

The observed benefits are biologically plausible and align with the distinct properties of these DNA-derived polymers [1]. While PDRN is pharmacologically linked to tissue-regenerative signaling, modulation of inflammation, and angiogenesis [4, 5], PN acts as a physical scaffold [2, 3]. By creating an optimal three-dimensional environment, PN mechanically supports fibroblast activity and natural tissue restoration [7, 8]. Earlier use appeared to be associated with better outcomes in our series, particularly when DOT-PN was introduced during the inflammatory or early proliferative phase before extensive scar maturation or tissue loss had occurred.

A practical point raised by the reviewers is how PN differs from standard alternatives such as intralesional corticosteroid injections or other wound healing products. Corticosteroids remain a mainstay for established hypertrophic scars because they suppress fibroblast activity and collagen deposition. However, they may also worsen dermal atrophy, telangiectasia, and hypopigmentation, and they are less attractive when tissue fragility, ischemia, or volume deficiency is present. By contrast, PN is used with the goal of supporting scaffold-based tissue recovery and scar remodeling while preserving or improving local tissue quality rather than suppressing it.

Compared with conventional wound healing products such as silicone-based scar therapy, dressings, growth factor preparations, platelet-rich plasma, or hyaluronic acid-based injectables, PN offers an injectable regenerative approach that can be targeted directly to compromised postoperative tissue. In our practice, this was particularly relevant when the clinical problem involved a combination of delayed healing and soft-tissue quality loss. PN should not be interpreted as a replacement for debridement, infection control, tension reduction, vascular assessment, or established scar therapies; instead, it may serve as a biologically oriented adjunct in selected patients.

This study has important limitations. It was retrospective, involved a small sample, and included heterogeneous indications and treatment schedules. Outcome assessment relied mainly on clinical examination and serial photography rather than standardized validated scales. The distinction between PN and PDRN is also relevant because terminology in aesthetic medicine remains evolving, and recent work has emphasized the need for standardized nomenclature [10]. In addition, the current report is descriptive and does not establish comparative efficacy against corticosteroids, silicone therapy, platelet-rich plasma, or other wound healing interventions. Larger prospective studies using predefined protocols and validated scar instruments are needed.

## Conclusion

Injectable PN therapy appears to be a safe and clinically useful adjunct for wound repair and scar modulation after aesthetic procedures. In this retrospective case series, the treatment was associated with improved healing, scar quality, and contour restoration, especially when initiated early in high-risk wounds. Prospective controlled studies should clarify patient selection, standardized dosing, comparative effectiveness, and long-term outcomes.
